# Preferred conditions for promoting participation in on-site oral health surveys among Japanese adults: insights from a conjoint analysis

**DOI:** 10.1186/s12889-026-26736-3

**Published:** 2026-04-06

**Authors:** Katsuo Oshima, Hideki Fukuda, Yukie Murata, Yusuke Ida, Yoichi Ishizuka, Hiroko Miura

**Affiliations:** 1 Department of Oral Health, School of Life Dentistry at Tokyo, The Nippon Dental University, 1-9-20 Fujimi, Chiyoda-ku, Tokyo, 102-8159 Japan; 2 National Institute of Public Health, 2-3-6 Minami, Wako-shi, Saitama, 351-0197 Japan; 3 Division of Disease Control and Epidemiology, School of Dentistry, Health Sciences University of Hokkaido, 1757 Kanazawa, Tobetsu-cho, Ishikari-gun, Hokkaido, 061-0293 Japan; 4 Department of Healthcare Information Management, The University of Tokyo Hospital, 7-3-1 Hongo, Bunkyo-ku, Tokyo, 113-0033 Japan; 5 Department of Epidemiology and Public Health, Tokyo Dental College, 2-9-18 Kandamisaki-cho, Chiyoda-ku, Tokyo, 101-0061 Japan

**Keywords:** Oral health survey, Conjoint analysis, Survey participation, Urban-rural differences, Japan

## Abstract

**Background:**

On-site oral health surveys conducted to epidemiologically assess dental diseases require strategies to prevent a decline in the number of participants and maintain data quality; however, few studies have examined measures to address this issue. This study aimed to identify the preferred conditions for promoting participation in on-site oral health surveys using conjoint analysis.

**Methods:**

A cross-sectional study using an online survey was conducted in Japan in January 2024. From a panel of an online research company, 1,260 individuals (420 residents of urban, intermediate, and rural areas) were randomly sampled. The participants were evaluated under 16 hypothetical scenarios for oral health surveys, each consisting of seven attributes and their respective levels. Monetary incentives were excluded as attributes, because such measures are typically not applicable to government-administered surveys.

**Results:**

In total, 955 individuals were analysed (324 in urban areas; 333 in intermediate areas; and 298 in rural areas). Our conjoint analysis showed that in all three areas, the preferred conditions to promote participation in oral health surveys were nearby dental clinics, conducting the survey on a Saturday or Sunday, providing an explanation of oral health status by a dentist after the survey, and provision of small gifts such as toothbrushes. Conversely, the following conditions were rated unfavourably: location–home visits by investigators, and day of the week–weekdays. The modified Poisson regression analysis revealed that those who did not intend to participate in all 16 hypothetical scenarios were statistically significantly more likely to be older in urban and intermediate areas (prevalence ratio, urban, 60–69 years: 2.52; intermediate, 60–69 years: 2.19).

**Conclusions:**

Our findings suggest that an effective strategy to promote participation in on-site oral health surveys includes conducting a survey at nearby dental clinics on holidays, providing participants with an explanation of their oral health status by a dentist, and distributing small gifts such as toothbrushes after the survey. In addition, targeted approaches may be required to encourage participation among older adults, particularly in urban and intermediate areas.

**Supplementary Information:**

The online version contains supplementary material available at 10.1186/s12889-026-26736-3.

## Background

Official epidemiological oral health surveys conducted across countries are essential to assess population oral health status and to inform the development of oral health policies [1–5]. However, declining participation rates threaten data quality and hinder policy planning, a challenge observed across various survey types and health domains worldwide.

In Japan, the Ministry of Health, Labour and Welfare (MHLW) has conducted the Survey of Dental Diseases since 1957, with subsequent rounds conducted at multi-year intervals; the 13th survey was completed in 2024 [1]. In this national oral health survey, Japanese residents were invited to designated venues, where dentists conducted examinations to assess dental diseases such as dental caries and periodontal disease. The survey employed stratified random sampling of residents across Japan; however, participation has declined with each successive round. If this trend persists, the survey results may lose national representativeness, limiting the utility for oral health policy planning. Consequently, strategies to enhance public participation in such surveys should be considered.

Previous studies suggest that individuals who participate in on-site oral health surveys are likely to be unemployed and regularly visit dental clinics [6, 7]. Common reasons for nonparticipation include difficulty securing time due to long working hours and the distance of the survey venue from home [8]. This trend is not unique to oral health surveys, but is also observed in general health surveys, suggesting that people prefer greater flexibility in choosing the time and location of their health examinations [9, 10]. In addition, factors such as old age and physical ailments are barriers to participation [11]. The decline in participation rates is a widespread issue in many countries, and various discussions are underway to address this challenge [12–15].

Epidemiological studies employ various methods, including face-to-face interviews, postal questionnaires, and online surveys, and studies support the use of monetary incentives to increase participation rates [16–18]. Among these, cash incentives are more effective than vouchers or lotteries [16]. Therefore, provision of monetary incentives to survey participants can be considered a highly effective measure to promote participation in on-site oral health surveys. However, these are costly, and given that Japan’s Survey of Dental Diseases is conducted by government agencies and funded by public taxes, offering such incentives is not feasible. Thus, the identification of optimal conditions for promoting participation in on-site oral health surveys is required, considering realistically feasible conditions; however, no such studies have been conducted.

We, therefore, aimed to conduct a conjoint analysis survey targeting Japanese residents to identify the conditions most preferred for participating in on-site oral health surveys, and to evaluate the characteristics of individuals who were unwilling to participate. Our findings are expected to inform strategies for improved participation in future on-site oral health surveys.

## Methods

### Design, setting, and participants

This cross-sectional study used an online survey conducted from 12 to 15 January 2024 by a Japanese online research company (Macromill, Inc., Tokyo) as part of a research project commissioned by the MHLW. This research company has approximately 1.3 million registered panel members, equivalent to approximately 1% of the Japanese population [19].

Participants were randomly sampled from a registered panel of the research company using the quota sampling method, and stratified by municipality of residence, sex, and age, as described previously [20]. As conditions for quota sampling, we first classified municipalities of residence into the following three groups: (1) urban areas, designated cities with a population of ≥ 500,000; (2) intermediate areas, cities with a population of ≥ 100,000, excluding designated cities; and (3) rural areas, cities with a population of < 100,000, towns, and villages. For each group of municipalities, we set equal proportions for gender (male/female) and age (stratified by decade) and then sampled men and women aged 20–69 years.

Regarding sample size, previous studies using conjoint analysis surveys typically included approximately 300 participants [21, 22]. In this study, participants who responded with the same value on the Likert scale for the 16 hypothetical scenarios of oral health surveys were excluded from the statistical analysis. However, predicting the number of exclusions in advance is difficult. Therefore, to obtain a sufficient sample size within the available research budget, the target sample size for each regional area was set at 400 individuals. Ultimately, 420 residents from each regional area participated, for a total of 1,260 participants.

## Conjoint analysis survey

To create a questionnaire for the conjoint analysis survey, we defined the attributes and levels likely to influence participation in an on-site oral health survey. These attributes and levels were selected based on discussions among public health researchers (six authors) with reference to previous studies [6–9, 15]. In addition, we interviewed two prefectural government officials to assess the content validity of the questionnaire items, as the Japanese Survey of Dental Diseases is administered by the MHLW but its implementation is delegated to prefectural governments [1].

The attributes and levels that constitute the hypothetical scenario for the oral health survey were set as follows: (1) location, including public facilities such as community and health centres, nearby dental clinics, commercial facilities such as supermarkets and shopping malls, and home visits by an investigator; (2) day of the week, including early weekdays (Monday to Wednesday), later weekdays (Thursday and Friday), Saturday, and Sunday; (3) time required for the survey, as short as possible, acceptable even if it takes some time; (4) advance booking of survey time, available/not available; (5) explanation of oral health status by dentist after survey (provided/not provided); (6) small gifts such as toothbrushes (provided/not provided); (7) orientation session before the survey (held/not held) (Table 1). Of these seven attributes, attributes (1) and (2) had four levels, and attributes (3) to (7) had two levels; we created 16 combinations of hypothetical scenarios for oral health surveys based on an orthogonal table (L^16^(2^5^4^2^)) (Table 2). Monetary incentives were excluded because they were not feasible.

**Table 1:** Attributes and levels included in the conjoint analysis survey

Attributes	Levels
Location	Public facilities (community centres, health centres, etc.) Nearby dental clinics Commercial facilities (supermarkets, shopping malls, etc.) Home visits by investigators
Day of the week	Early weekdays (Monday–Wednesday) Later weekdays (Thursday and Friday) Saturday Sunday
Time required for the survey	As short as possible Acceptable even if it takes some time
Advance booking of survey time	Available Not available
Explanation of oral health status by dentist after survey	Provided Not provided
Small gifts (toothbrushes, etc.)	Provided Not provided
Orientation session before the survey	Held Not held

**Table 2:** Types of hypothetical scenarios for oral health surveys

No	Location	Day of the week	Time required for the survey	Advance booking of survey time	Explanation of oral health status by dentist after survey	Small gifts	Orientation session before the survey
1	Public facilities	Early weekdays (Monday–Wednesday)	As short as possible	Available	Provided	Provided	Held
2	Public facilities	Later weekdays (Thursday and Friday)	As short as possible	Available	Provided	Not provided	Not held
3	Public facilities	Saturday	Acceptable even if it takes some time	Not available	Not provided	Provided	Held
4	Public facilities	Sunday	Acceptable even if it takes some time	Not available	Not provided	Not provided	Not held
5	Nearby dental clinics	Early weekdays (Monday–Wednesday)	As short as possible	Not available	Not provided	Provided	Not held
6	Nearby dental clinics	Later weekdays (Thursday and Friday)	As short as possible	Not available	Not provided	Not provided	Held
7	Nearby dental clinics	Saturday	Acceptable even if it takes some time	Available	Provided	Provided	Not held
8	Nearby dental clinics	Sunday	Acceptable even if it takes some time	Available	Provided	Not provided	Held
9	Commercial facilities	Early weekdays (Monday–Wednesday)	Acceptable even if it takes some time	Available	Not provided	Not provided	Held
10	Commercial facilities	Later weekdays (Thursday and Friday)	Acceptable even if it takes some time	Available	Not provided	Provided	Not held
11	Commercial facilities	Saturday	As short as possible	Not available	Provided	Not provided	Held
12	Commercial facilities	Sunday	As short as possible	Not available	Provided	Provided	Not held
13	Home	Early weekdays (Monday–Wednesday)	Acceptable even if it takes some time	Not available	Provided	Not provided	Not held
14	Home	Later weekdays (Thursday and Friday)	Acceptable even if it takes some time	Not available	Provided	Provided	Held
15	Home	Saturday	As short as possible	Available	Not provided	Not provided	Not held
16	Home	Sunday	As short as possible	Available	Not provided	Provided	Held

Before conducting the conjoint analysis survey, the survey participants were first presented with an explanation of the current Survey of Dental Diseases in Japan. The written explanation was as follows: “One of the surveys conducted by the Ministry of Health, Labour and Welfare is the Survey of Dental Disease. The purpose of this survey is to understand the dental and oral health status of people living in Japan and collect basic data for developing dental health policies. To conduct the survey, several regions across Japan were selected and residents in those areas were invited to participate. Selected individuals visit venues such as community centres where dentists conduct dental examinations. However, in recent years, the number of participants in this survey has declined, which has become a serious concern.”

Next, participants were asked to select the score that best reflected their willingness to participate in each of 16 hypothetical oral health survey scenarios using an 11-point Likert scale ranging from 0 to 10 (0, Not willing to participate; 5, Neither willing nor unwilling to participate; 10, Willing to participate). Sixteen hypothetical scenarios were randomly displayed. Participants were required to respond before proceeding to the next screen to avoid missing values. The questionnaire is presented in Additional File 1 (Supplementary Materials).

### Variables related to individual characteristics of participants

The variables related to participant characteristics included socioeconomic status and oral health status. All variables were treated as categorical variables. Regarding variables related to socioeconomic status, we stratified by sex, age, household income, marital status, and working status. Participants were classified into five age groups: 20–29, 30–39, 40–49, 50–59, and 60–69 years. Household income was classified into six groups: JPY < 2 million, JPY 2–4 million, JPY 4–6 million, JPY 6–8 million, JPY ≥ 8 million, and unknown (as of 2022, the median was 4.23 million yen [23]). Marital status was classified as married or unmarried. Working status was classified into four groups: regular worker, homemaker, part-time worker, and unemployed or other.

Regarding oral health status variables, we stratified by frequency of tooth brushing and regularity of dental check-ups. The frequency of brushing teeth was classified into four groups: ≥three times daily, twice daily, once daily, and sometimes/no brushing. Status of receiving regular dental check-ups were categorised as yes or no. In addition, participants were asked whether they had previously participated in the Survey of Dental Diseases conducted by the MHLW.

### Statistical analysis

Prior to the statistical analysis, study participants who responded with the same values on the Likert scale for all 16 hypothetical oral health survey scenarios were excluded as exhibiting invalid responses due to straightlining [24, 25]. As a result, the analysis population in this study included 324 individuals from urban areas, 333 individuals from intermediate areas, and 298 individuals from rural areas, totalling 955 individuals (Table 3). In addition, we compared demographic characteristics between the analysis and excluded populations using chi-square tests.

**Table 3:** Study participants and analysis populations

Urban area	Study participants (a)	Those who responded with the same values on the Likert scale for all 16 hypothetical scenarios (b)	Analysis population (a-b)
	420	96	324
Intermediate area	420	87	333
Rural area	420	122	298
Total	1,260	305	955

To understand demographic characteristics of the analysis population, descriptive statistics were calculated for each variable related to socioeconomic status and oral health status.

Thereafter, to evaluate preferred conditions for an on-site oral health survey based on willingness to participate ratings for 16 hypothetical scenarios (0–10 points), we conducted a rating-based conjoint analysis stratified by three residential areas (urban, intermediate, and rural) and calculated relative importance and part-worth utility values. Herein, relative importance represents the proportional contribution of seven attributes—“location,” “day of the week,” “time required for the survey,” “advance booking of survey time,” “explanation of oral-health status by dentist after survey,” “small gifts,” and “orientation session before the survey”—to the willingness to participate ratings; a larger value indicates a greater importance of the attribute. Part-worth utility values indicate the relative contribution of each level within an attribute to the ratings; levels with larger (more positive) utilities contribute more to higher willingness to participate ratings within that attribute. Part-worth utility values were derived from the coefficients obtained by an ordinary least squares (OLS) regression model, with the rating for each hypothetical scenario as the dependent variable and the experimental design (i.e., the combination of attribute levels) as independent variables, where attribute levels were entered as dummy variables. Relative importance was calculated by computing the range (maximum minus minimum) of the part-worth utility values for each attribute, dividing it by the sum of the ranges across all attributes, and expressing the result as a percentage. To assess model goodness-of-fit, we calculated Pearson’s correlation coefficient between the observed mean rating for each of the 16 scenarios and the corresponding predicted value from the OLS model for each area. This coefficient indicates the extent to which the model reproduces the scenario-specific pattern of mean ratings.

Furthermore, across all 16 hypothetical scenarios, respondents who assigned a score of 0–5 on the 11-point Likert scale (0–10) were categorised as individuals who did not express a clear intention (i.e., positive willingness) to participate in the oral health survey, and their characteristics were examined. In this study, scores of 6–10 were interpreted as indicating clear willingness to participate, whereas scores of 0–5—including negative and neutral responses—were defined as representing the absence of a positive participation intention, consistent with an implementation-oriented distinction between those who are clearly willing to participate and all other respondents. The proportion of respondents classified in this manner exceeded 10% in all three regions (urban: 21.6%; intermediate: 20.7%; rural: 19.8%) (Additional File 2). Therefore, we used modified Poisson regression analysis with robust error variance to identify factors (socioeconomic status and oral health status) associated with the absence of a positive participation intention [26]. Modified Poisson regression models were fitted using univariate analyses and multivariable analyses with forced entry, and prevalence ratio (PR) with 95% confidence interval (95% CI) were estimated separately for each region. The binary outcome variable was coded as 1 for respondents classified as having an absence of positive participation intention (scores 0–5) and as 0 for respondents who clearly indicated willingness to participate (scores 6–10).

Statistical analyses were performed using Stata version 18 (StataCorp LLC, College Station, TX). The statistical significance level was set at 5%.

### Ethical considerations

This study was conducted in accordance with the principles of the Declaration of Helsinki and the Ethical Guidelines for Medical and Biological Research Involving Human Subjects established by Japanese Government Agencies. This study was approved by the Ethics Review Board of the Nippon Dental University College at Tokyo (November 2023; #313). Informed consent was obtained from all the participants via a website. Participants’ personal information was protected by the research company.

## Results

### Demographic characteristics classified by municipality type

Table 4 presents the demographic characteristics of the analysis population in each regional area. The population comprised 324 urban residents (47.8% males), 333 intermediate residents (48.9% males), and 298 rural residents (47.7% males). Of the respondents who reported prior participation in the Survey of Dental Diseases conducted by the MHLW, three were from urban areas, eight were from intermediate areas, and seven were from rural areas. Because their inclusion was deemed unlikely to bias the results, they were retained in the analysis population.

**Table 4:** Demographic characteristics of the analysis population, classified by municipality type

	Urban (*n* = 324)		Intermediate (*n* = 333)		Rural (*n* = 298)	
	*n*	(%)	*n*	(%)	*n*	(%)
Gender						
Male	155	(47.8)	163	(48.9)	142	(47.7)
Female	169	(52.2)	170	(51.1)	156	(52.3)
Age						
20–29 years	69	(21.3)	69	(20.7)	64	(21.5)
30–39 years	70	(21.6)	63	(18.9)	65	(21.8)
40–49 years	65	(20.1)	73	(21.9)	55	(18.5)
50–59 years	65	(20.1)	65	(19.5)	59	(19.8)
60–69 years	55	(17.0)	63	(18.9)	55	(18.5)
Household income						
JPY < 2 million	27	(8.3)	21	(6.3)	27	(9.1)
JPY 2–4 million	49	(15.1)	61	(18.3)	53	(17.8)
JPY 4–6 million	52	(16.0)	63	(18.9)	71	(23.8)
JPY 6–8 million	40	(12.3)	48	(14.4)	31	(10.4)
JPY ≥ 8 million	60	(18.5)	55	(16.5)	36	(12.1)
Unknown	96	(29.6)	85	(25.5)	80	(26.8)
Marital status						
Married	175	(54.0)	193	(58.0)	182	(61.1)
Single	149	(46.0)	140	(42.0)	116	(38.9)
Working status						
Regular worker	178	(54.9)	189	(56.8)	167	(56.0)
Homemaker	54	(16.7)	38	(11.4)	41	(13.8)
Part-time worker	54	(16.7)	57	(17.1)	51	(17.1)
Unemployed/others	38	(11.7)	49	(14.7)	39	(13.1)
Frequency of brushing teeth						
≥Three times daily	81	(25.0)	86	(25.8)	71	(23.8)
Twice daily	176	(54.3)	172	(51.7)	159	(53.4)
Once daily	54	(16.7)	68	(20.4)	55	(18.5)
Sometimes/No brushing	13	(4.0)	7	(2.1)	13	(4.4)
Regular dental check-up						
Yes	166	(51.2)	169	(50.8)	155	(52.0)
No	158	(48.8)	164	(49.2)	143	(48.0)

Regarding the comparison between the analysis and excluded populations, no statistically significant differences were observed for basic characteristics, except for regular dental check-ups (Additional File 3).

### Relative importance of attributes in hypothetical scenarios

**Fig. 1:**
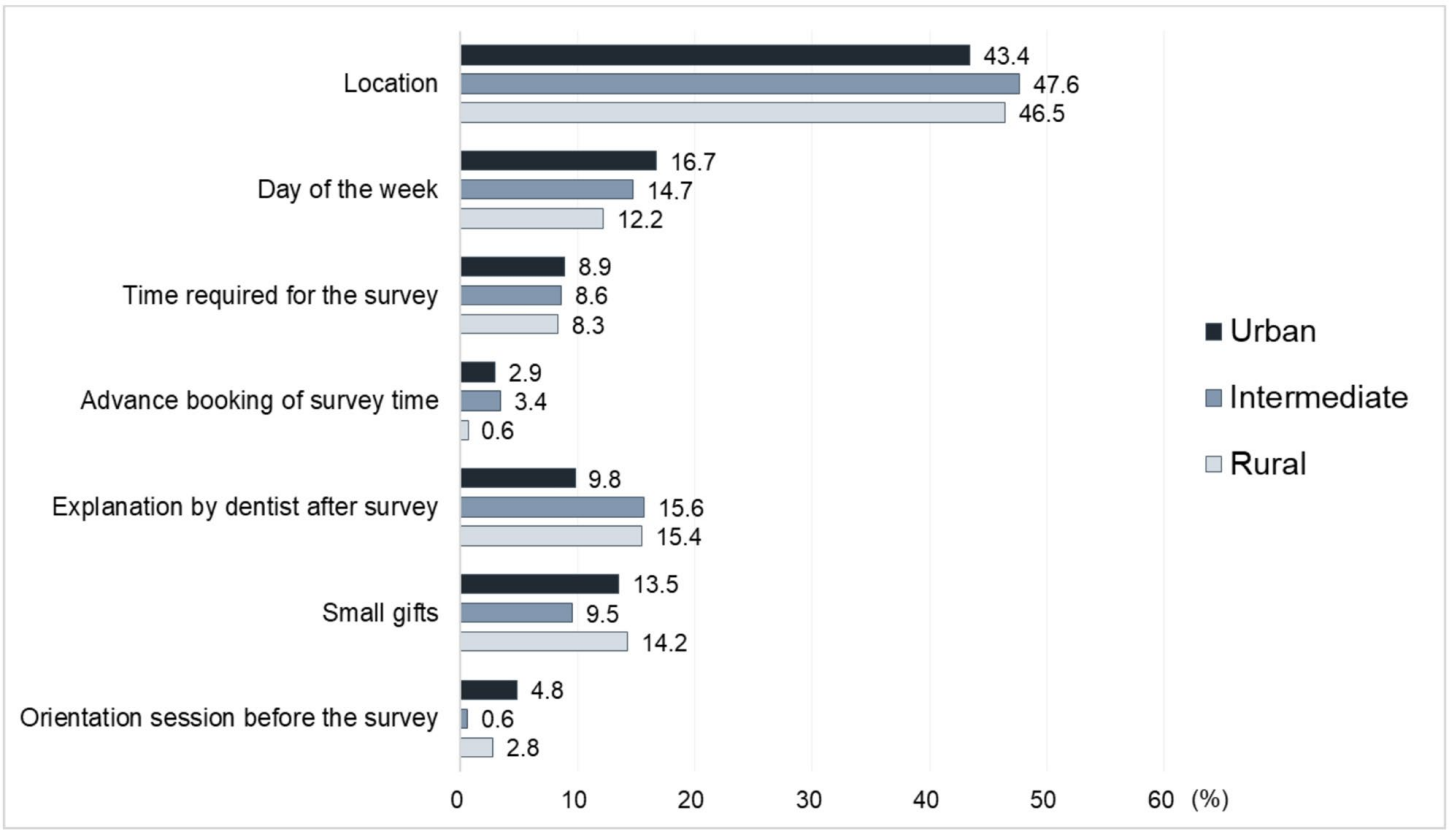
Relative importance of attributes in the hypothetical scenarios for oral health survey. Each attribute is classified by municipality type

Figure 1 shows the relative importance of each attribute derived from the conjoint analysis, based on evaluations of the hypothetical oral health survey scenarios provided by the analysis population stratified by regional area. In urban areas, the following attributes showed high proportions: location (43.4%), day of the week (16.7%), small gifts (13.5%), explanation of oral health status by dentist after survey (9.8%), time required for the survey (8.9%), orientation session before the survey (4.8%), and advance booking of survey time (2.9%). In intermediate areas, the following attributes showed high proportions: location (47.6%), explanation of oral health status by dentist after survey (15.6%), day of the week (14.7%), small gifts (9.5%), time required for the survey (8.6%), advance booking of survey time (3.4%), and orientation session before the survey (0.6%). In rural areas, the following attributes showed high proportions: location (46.5%), explanation of oral health status by dentist after survey (15.4%), small gifts (14.2%), day of the week (12.2%), time required for the survey (8.3%), orientation session before the survey (2.8%), and advance booking of survey time (0.6%) (details are presented in Additional File 4). The Pearson’s correlation coefficients were 0.991 (< 0.001) in urban areas, 0.995 (< 0.001) in intermediate areas, and 0.995 (< 0.001) in rural areas.

### Part-worth utility values

**Fig. 2:**
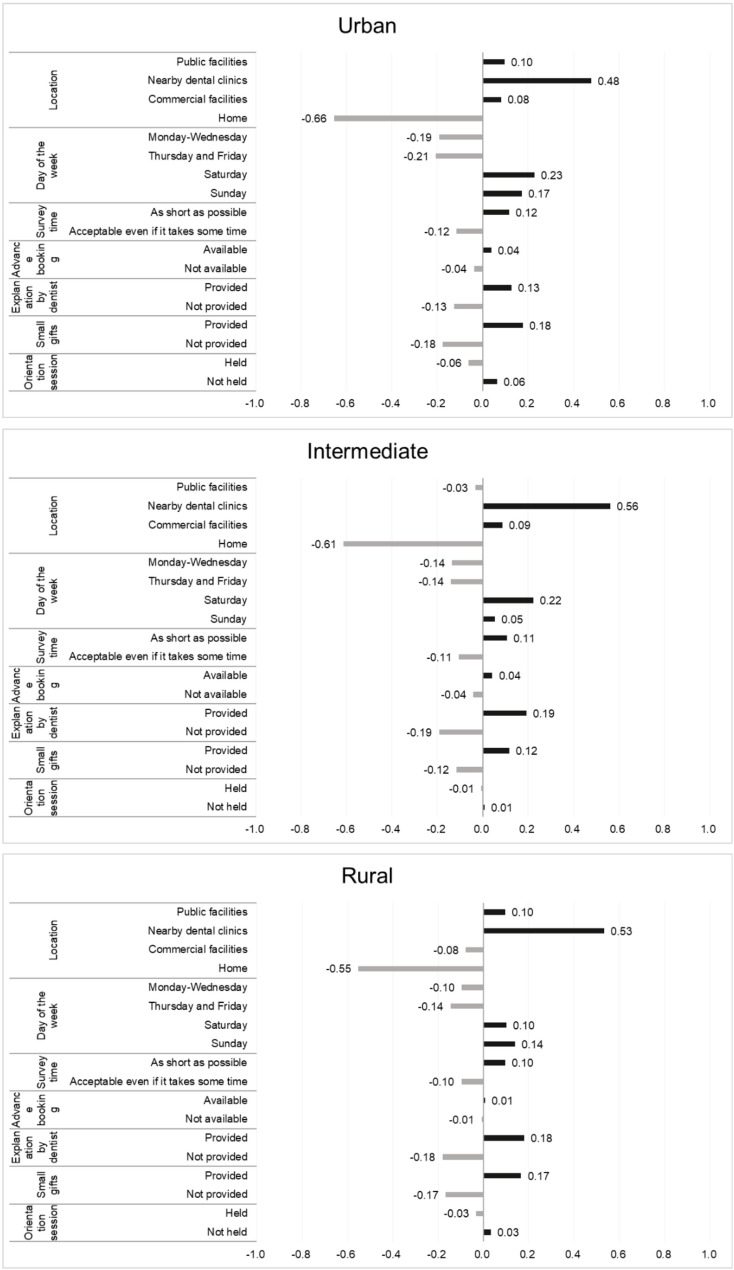
Utility values of each attribute and levels in the hypothetical scenarios. Each attribute is classified by municipality type

Figure 2 presents the part-worth utility values for each level within each attribute, estimated through a conjoint analysis based on evaluations of hypothetical oral health survey scenarios provided by the analysis population stratified by regional area. In urban areas, the following attribute-levels were preferred: location–nearby dental clinics (0.48), day of the week–Saturday (0.23), and small gifts–provided (0.18). In contrast, the following attribute-levels were rated unfavourably: location–home visits by investigators (-0.66) and day of the week–Monday to Wednesday (-0.19) and Thursday and Friday (-0.21). In intermediate areas, the following attribute-levels were preferred: location–nearby dental clinics (0.56), day of the week–Saturday (0.22), and explanation of oral status by dentist after survey–provided (0.19). In contrast, the following attribute-levels were rated unfavourably: location–home visits by investigators (-0.61), and day of the week–Monday to Wednesday (-0.14) and Thursday and Friday (-0.14). In rural areas, the following attribute-levels were particularly preferred: location–nearby dental clinics (0.53), explanation of oral status by dentist after survey–provided (0.18), and small gifts–provided (0.17). In contrast, the following attribute-levels was rated unfavourably: location–home visits by investigators (-0.55) (details are presented in Additional File 4).

### Characteristics of those who have no intention of participating in the oral-health survey

Table 5 shows the PR and 95% confidence interval (95%CI) calculated using modified Poisson regression analysis (multivariate analysis) to determine the association between the intention to participate in hypothetical oral-health survey scenarios (individuals categorised as having no intention to participate = 1; all other individuals = 0) and the variables related to individual characteristics. The results of the univariate modified Poisson regression analysis stratified by municipality type are presented in Additional File 5, and the proportion of individuals who did not intend to participate is shown in Additional File 2.

**Table 5:** Characteristics of those who did not intend to participate in the oral health survey, classified by municipality type (Multivariate modified Poisson regression analysis)

	Urban	Intermediate	Rural
	PR (95%CI)	PR (95%CI)	PR (95%CI)
Gender			
Male	1.17 (0.71–1.93)	0.77 (0.48–1.23)	1.27 (0.72–2.25)
Female	reference	reference	Reference
Age			
20–29 years	reference	reference	Reference
30–39 years	1.21 (0.59–2.51)	0.99 (0.41–2.36)	1.74 (0.82–3.67)
40–49 years	0.76 (0.33–1.75)	1.74 (0.86–3.53)	1.21 (0.52–2.81)
50–59 years	1.07 (0.51–2.22)	2.18 (1.11–4.28) *	1.27 (0.58–2.79)
60–69 years	2.52 (1.29–4.92) **	2.19 (1.08–4.41) *	1.64 (0.74–3.65)
Household income			
JPY < 2 million	reference	reference	Reference
JPY 2–4 million	1.56 (0.44–5.61)	0.53 (0.25–1.10)	1.14 (0.54–2.37)
JPY 4–6 million	1.93 (0.54–6.92)	0.42 (0.18–0.94) *	0.65 (0.29–1.46)
JPY 6–8 million	3.33 (0.96–11.55)	0.47 (0.18–1.24)	0.81 (0.33–1.98)
JPY ≥ 8 million	2.63 (0.74–9.33)	0.36 (0.15–0.89) *	0.50 (0.16–1.53)
Unknown	2.55 (0.76–8.59)	0.55 (0.27–1.11)	0.84 (0.41–1.69)
Marital status			
Married	0.63 (0.38–1.03)	1.23 (0.75–2.01)	1.11 (0.65–1.91)
Single	reference	reference	Reference
Working status			
Regular worker	reference	reference	Reference
Homemaker	1.23 (0.58–2.62)	0.26 (0.08–0.83) *	0.86 (0.34–2.17)
Part-time worker	1.55 (0.90–2.67)	0.92 (0.51–1.66)	0.90 (0.41–1.96)
Unemployed/others	0.52 (0.20–1.32)	1.11 (0.63–1.98)	1.32 (0.66–2.63)
Frequency of brushing teeth			
≥Three times daily	0.86 (0.42–1.77)	0.78 (0.42–1.45)	1.28 (0.51–3.26)
Twice daily	1.29 (0.72–2.33)	0.76 (0.45–1.27)	2.21 (1.05–4.62) *
Once daily	reference	reference	reference
Sometimes/No brushing	1.27 (0.52–3.09)	1.68 (0.59–4.79)	2.90 (1.20–6.97) *
Regular dental check-up			
Yes	0.89 (0.58–1.36)	0.64 (0.42–0.98) *	0.68 (0.42–1.10)
No	reference	reference	reference

*PR* Prevalence ratio, *95%CI* 95% confidence interval

* *p* < 0.05, ** *p* < 0.01

In multivariate analysis after adjusting for all variables, in urban areas, the PR was statistically significantly higher among those aged 60–69 years (PR [95%CI]) = 2.52 [1.29–4.92]). In the intermediate area, the PR was statistically significantly higher among those aged 50–59 years (2.18 [1.11–4.28]) and those aged 60–69 years (2.19 [1.08–4.41]); however, it was lower among those with higher household income (JPY 4–6 million: 0.42 [0.18–0.94], JPY ≥ 8 million: 0.36 [0.15–0.89]), homemakers (0.26 [0.08–0.83]), and those with receiving regular dental check-ups (0.64 [0.42–0.98]). In rural areas, the PR was statistically significantly higher for those who practiced teeth brushing (twice daily: 2.21 [1.05–4.62]), and sometimes/no brushing (2.90 [1.20–6.97]).

## Discussion

Our key findings were as follows: (1) across all three regional areas, the preferred conditions for encouraging participation included conducting the survey at nearby dental clinics, scheduling it on weekends (Saturdays or Sundays), providing an explanation of the oral health status by a dentist after the survey, and offering small gifts such as toothbrushes; (2) In urban and intermediate areas, older adults were more likely to not express a willingness to participate in all 16 hypothetical oral health survey scenarios.

The most influential factor in promoting participation in oral health surveys was survey location, a trend consistently observed across the urban, intermediate, and rural regions, with each accounting for approximately 45% of the relative importance. Among the levels of location attributes, respondents preferred conducting the survey at a nearby dental clinic. In terms of scheduling, weekends (Saturday or Sunday) were favoured over weekdays, suggesting that the study population preferred survey opportunities that better accommodated personal availability. These findings are consistent with those of previous studies, which have identified distant survey venues and limited time availability as major barriers to participation in health surveys [9, 10]. Taken together, these results suggest that potential survey population value efficiency seeks to minimise the time burden associated with survey participation.

Our participants favoured shorter survey durations, further underscoring their desire to minimise the time commitment required. Given that the study sample was limited to individuals aged 20 to 69 years—many of whom are likely to face time constraints owing to work and household responsibilities—these preferences appear to reflect the practical realities of their daily lives. Japan has achieved universal health coverage in dental care, ensuring free choice of dental clinics nationwide [27]. Although the proportion of individuals with a family dentist varies according to region and sex, it ranges from 40% to 50% [20, 28]. Furthermore, 63.8% of the participants underwent regular dental check-ups [1]. These figures suggest that dental clinics are familiar and accessible to Japanese residents. Accordingly, utilising dental clinics as venues for oral health surveys may represent a practical and effective strategy for enhancing participation.

Our participants preferred to receive an explanation of their oral health status from a dentist following the survey as well as small gifts such as toothbrushes. While studies have highlighted the importance of monetary incentives in promoting survey participation [16–18], we deliberately excluded monetary incentives. Consequently, the preferences may reflect alternative forms of incentives that participants find meaningful in the absence of monetary compensation. Furthermore, an implication of our findings is that the choice of survey venue was more influential in encouraging participation than post-survey incentives such as health feedback or small gifts. This suggests that although incentives are commonly emphasised as a strategy to enhance participation, attention should be paid to the selection of survey locations, especially in contexts where the provision of monetary incentives is not feasible.

Scheduling survey appointments in advance and holding pre-survey orientation sessions were not regarded as important by participants. Rather than reflecting a concern for time efficiency, this may indicate that preparatory tasks such as making appointments or attending an explanatory orientation are perceived as low-priority activities within the context of the participants’ daily lives. Additionally, the study revealed that the option of having investigators visit the participants’ homes to conduct the survey was the most strongly avoided. This aversion may reflect the low prioritisation of oral health surveys, particularly when they intrude upon private spaces such as the home.

When comparing the urban, intermediate, and rural areas, the results of the conjoint analysis revealed similar trends across all regions. When viewed from the perspective of conditions that promote participation in on-site oral health surveys, the preferred conditions did not differ by region. Nevertheless, the existing literature has generally reported regional disparities between urban and rural areas in oral health-related factors such as access to dental healthcare service, with rural areas facing greater barriers [20, 29–32]. Furthermore, previous studies examining regional differences in research participation have shown that rural areas tend to have fewer participants than urban areas [33, 34]. In the present study, conditions that should be considered by those organising on-site oral health surveys were identified. However, from the perspective of participant access to survey venues by region, further research is warranted.

Furthermore, according to the results of our analysis conducted to identify factors associated with individuals who did not express willingness to participate in any of the 16 hypothetical oral health survey scenarios set in this study, a higher proportion of older adults was observed, particularly in urban and intermediate areas. In the intermediate area, multivariable analysis revealed that for other variables, there was a statistically significant lower prevalence of individuals with higher household incomes and those undergoing regular dental check-ups; thus, the low affinity toward dental visits may have been associated with the lack of expressed willingness to participate in the survey. In urban areas, although no statistically significant associations were found, there was a tendency toward fewer individuals receiving regular dental check-ups. Physical vulnerabilities, such as advanced age and physical illness, can act as a barrier to participation in health surveys [9, 11]. The older adults identified in our multivariate analysis were those aged 60–69 years in urban areas and 50–69 years in intermediate areas; it remains unclear whether the responses from these individuals were due to physical illness. Nevertheless, measures targeting older adults are indispensable to promote participation in oral health surveys, and our study provides data supporting this necessity.

This study has some limitations. First, as this study employed a cross-sectional design and analysed data collected at a single time point, it was not possible to identify any causal relationships between the preferred conditions for promoting participation in oral health surveys and the individual characteristics of the study participants. Second, the participants were recruited from a registered panel of an online research company, which may have introduced selection bias. Although Internet usage has been increasing in Japan [35], and the research company maintains a large panel of approximately 1.3 million individuals, these participants do not represent the entire Japanese population. Third, most participants had no prior experience with surveys of dental diseases in Japan. While an overview of the survey was presented on screen before the conjoint analysis survey, information bias arising from insufficient familiarity with the actual survey procedures cannot be ruled out. Fourth, approximately 300 respondents who gave identical Likert scale ratings across all 16 hypothetical scenarios were excluded from the analysis, resulting in a substantial loss of individual‑level data. Such “straightlining” responses are generally regarded as invalid [24, 25], and future surveys should incorporate measures to minimise the occurrence of this behaviour. Fifth, this study employed a ratings-based conjoint analysis approach, which can efficiently quantify the relative importance of multiple survey attributes and the trade-offs among them [36, 37]. However, ratings on an 11-point scale may be influenced by individual differences in scale use (e.g., systematically higher or lower ratings), and information bias caused by the hypothetical nature of the scenarios cannot be ruled out [36, 37]. Accordingly, the findings may not perfectly translate into actual participation behaviour and should be interpreted with caution. Sixth, the study sample included only individuals aged 20–69 years, as those aged 70 and above were excluded due to the nature of the online survey. Advanced age is a known barrier to participating in health surveys [9, 11]. However, this study was unable to capture the circumstances of individuals aged 70 years and above and did not address strategies to promote their participation.

Despite these limitations, our findings demonstrate a degree of generalisability, particularly regarding the conditions required to implement on-site oral health surveys. We identified four key factors: location, day of the week, professional explanations after the survey, and participation in small-gift purchases. Considering Japan’s rapidly ageing population, it is essential to develop measures to promote survey participation among older adults requiring long-term care and those with disabilities. Future research should investigate and evaluate strategies aimed at facilitating oral health survey participation among vulnerable groups.

## Conclusions

We conducted a conjoint analysis to identify conditions that effectively promoted participation in on-site oral health surveys among online panel members aged 20–69 years. Across all three regions, the preferred conditions for participation included conducting surveys at nearby dental clinics, scheduling them on weekends, providing a dentist’s explanation of oral health status, and offering small gifts such as toothbrushes. Conversely, survey conditions involving home visits by investigators and weekday scheduling were rated unfavourably. Furthermore, in urban and intermediate regions, older (60–69 years) individuals were more likely to decline their participation across all hypothetical survey scenarios, suggesting that age-related factors influence their willingness to engage. These findings offer practical insights for designing oral health survey strategies tailored to digitally connected, working‑age populations, but may not be generalisable to adults aged ≥ 70 years or to those who are not digitally connected, highlighting a potential need for further investigation. 

## Supplementary Information

Supplementary Material 1.

## Data Availability

The datasets generated and/or analysed during the current study are not publicly available owing to participant privacy; however, they are available from the corresponding author upon reasonable request.

## Abbreviations

CI: Confidence interval
MHLW: Ministry of Health, Labour and Welfare
PR: Prevalence ratios

## References

[1] Ministry of Health, Labour and Welfare. Survey of dental diseases. https://www.mhlw.go.jp/toukei/list/62-17.html. Accessed 20 Jun 2025.

[2] UK GOV. Adult oral health survey 2021. https://www.gov.uk/government/statistics/adult-oral-health-survey-2021. Accessed 21 Jun 2025.

[3] Centers for Disease Control and Prevention. National health and nutrition examination survey. https://www.cdc.gov/nchs/nhanes/index.html. Accessed 21 Jun 2025.

[4] Statistics Canada. Canadian Health Measures Survey (CHMS). https://www.statcan.gc.ca/en/survey/household/5071. Accessed 21 Jun 2025.

[5] Australian Institute of Health and Welfare. National survey of adult Oral Health. https://www.aihw.gov.au/reports/dental-oral-health/oral-health-and-dental-care-in-australia/contents/data-sources. Accessed 21 Jun 2025.

[6] Matthews DC, Brillant MGS, Clovis JB, McNally ME, Filiaggi MJ, Kotzer RD, et al. Assessing the oral health of an ageing population: methods, challenges and predictors of survey participation. Gerodontology. 2012;29:e656–66. 21916953 10.1111/j.1741-2358.2011.00540.x PMC3499687

[7] Ando Y, Ikeda N, Nishi N, Tano R, Iwasaki M, Miura H. Assessment of participation and its associated lifestyle factors in the 2016 National Survey of Dental Diseases: an analysis through record linkage with National Health and Nutrition Survey. Nihon Koshu Eisei Zasshi. 2021;68:33–41. Japanese. 33342934 10.11236/jph.20-085

[8] Norheim PW, Helöe LA. Comparison between participants and non-participants in a dental health survey in northern Norway. Community Dent Oral Epidemiol. 1975;3:56–60. 1056287 10.1111/j.1600-0528.1975.tb00280.x

[9] Tolonen H, Lundqvist A, Jääskeläinen T, Koskinen S, Koponen P. Reasons for non-participation and ways to enhance participation in health examination surveys-the Health 2011 Survey. Eur J Public Health. 2017;27:909–11. 28957480 10.1093/eurpub/ckx098

[10] Reinikainen J, Saarsalmi P, Härkänen T, Jousilahti P, Karvanen J, Männistö S, et al. Non-participation modestly increased with distance to the examination clinic among adults in Finnish health examination surveys. Scand J Public Health. 2018;46:752–4. 29143578 10.1177/1403494817739502

[11] Gaertner B, Seitz I, Fuchs J, Busch MA, Holzhausen M, Martus P, et al. Baseline participation in a health examination survey of the population 65 years and older: who is missed and why? BMC Geriatr. 2016;16:21. 26787444 10.1186/s12877-016-0185-6 PMC4719664

[12] Mindell JS, Giampaoli S, Goesswald A, Kamtsiuris P, Mann C, Männistö S, et al. Sample selection, recruitment and participation rates in health examination surveys in Europe–experience from seven national surveys. BMC Med Res Methodol. 2015;15:78. 26438235 10.1186/s12874-015-0072-4 PMC4595185

[13] Lyshol H, Gil AP, Tolonen H, Namorado S, Kislaya I, Barreto M, et al. Local problem solving in the Portuguese health examination survey: a mixed method study. Arch Public Health. 2022;80:198. 36002860 10.1186/s13690-022-00939-7 PMC9400230

[14] Ishikawa M, Yokoyama T, Takimoto H. Possible measures to improve both participation and response quality in Japan’s national health and nutrition survey: results from a workshop by Local Government personnel in charge of the survey. Nutrients. 2022;14:3906. 36235557 10.3390/nu14193906 PMC9571403

[15] Ishikawa M, Hemmi O, Wada Y, Ohmi K, Ando Y, Takimoto H, et al. Efforts and systems by local governments to improve participation rates in national and local health and nutrition surveys in Japan: findings from a workshop 2019–2024. PLoS ONE. 2025;20:e0314798. 40029849 10.1371/journal.pone.0314798 PMC11875341

[16] Abdelazeem B, Hamdallah A, Rizk MA, Abbas KS, El-Shahat NA, Manasrah N, et al. Does usage of monetary incentive impact the involvement in surveys? A systematic review and meta-analysis of 46 randomized controlled trials. PLoS ONE. 2023;18:e0279128. 36649255 10.1371/journal.pone.0279128 PMC9844858

[17] Jia P, Furuya-Kanamori L, Qin ZS, Jia PY, Xu C. Association between response rates and monetary incentives in sample study: a systematic review and meta-analysis. Postgrad Med J. 2021;97:501–10. 32848082 10.1136/postgradmedj-2020-137868

[18] Edwards PJ, Roberts I, Clarke MJ, DiGuiseppi C, Woolf B, Perkins C. Methods to increase response to postal and electronic questionnaires. Cochrane Database Syst Rev. 2023;11:MR000008. 38032037 10.1002/14651858.MR000008.pub5 PMC10687884

[19] Ministry of internal affairs and communications population census. https://www.stat.go.jp/english/data/kokusei/index.html. Accessed 29 July 2025.

[20] Oshima K, Miura H, Tano R, Fukuda H. Urban-rural differences in the prevalence of having a family dentist and their association with income inequality among Japanese individuals: a cross-sectional study. BMC Oral Health. 2024;24:741. 38937717 10.1186/s12903-024-04528-8 PMC11210090

[21] Reed Johnson F, Lancsar E, Marshall D, Kilambi V, Mühlbacher A, Regier DA, et al. Constructing experimental designs for discrete-choice experiments: report of the ISPOR Conjoint Analysis Experimental Design Good Research Practices Task Force. Value Health. 2013;16:3–13. 23337210 10.1016/j.jval.2012.08.2223

[22] Larsen A, Tele A, Kumar M. Mental health service preferences of patients and providers: a scoping review of conjoint analysis and discrete choice experiments from global public health literature over the last 20 years (1999–2019). BMC Health Serv Res. 2021;21:589. 34144685 10.1186/s12913-021-06499-w PMC8214295

[23] Ministry of Health, Labour and Welfare. Comprehensive survey of living conditions. https://www.mhlw.go.jp/toukei/list/20-21kekka.html. Accessed 20 Jun 2025.

[24] Kim Y, Dykema J, Stevenson J, Black P, Moberg DP, Straightlining. Overview of measurement, comparison of indicators, and effects in mail–web mixed-mode surveys. Social Sci Comput Rev. 2019;37:214–33.

[25] Mirzaei A, Carter SR, Patanwala AE, Schneider CR. Missing data in surveys: Key concepts, approaches, and applications. Res Social Adm Pharm. 2022;18:2308–16. 33775556 10.1016/j.sapharm.2021.03.009

[26] Zou G. A modified poisson regression approach to prospective studies with binary data. Am J Epidemiol. 2004;159:702–6. 15033648 10.1093/aje/kwh090

[27] Ministry of Health, Labour and Welfare. Health insurance. https://www.mhlw.go.jp/english/policy/health-medical/health-insurance/index.html. Accessed 18 Aug 2025.

[28] Oshima K, Miura H, Tano R, Fukuda H. Characteristics of individuals in Japan who regularly manage their oral health by having a family dentist: A nationwide cross-sectional web-based survey. Int J Environ Res Public Health. 2022;19:10479. 36078196 10.3390/ijerph191710479 PMC9518108

[29] Alhozgi A, Feine JS, Tanwir F, Shrivastava R, Galarneau C, Emami E. Rural-urban disparities in patient satisfaction with oral health care: a provincial survey. BMC Oral Health. 2021;21:261. 33992110 10.1186/s12903-021-01613-0 PMC8122552

[30] Luo H, Wu Q, Bell RA, Wright W, Quandt SA, Basu R, et al. Rural-urban differences in dental service utilization and dental service procedures received among US adults: results from the 2016 medical expenditure panel survey. J Rural Health. 2021;37:655–66. 32697007 10.1111/jrh.12500 PMC7855605

[31] Crocombe LA, Chrisopoulos S, Kapellas K, Brennan D, Luzzi L, Khan S. Access to dental care barriers and poor clinical oral health in Australian regional populations. Aust Dent J. 2022;67:344–51. 35765724 10.1111/adj.12930 PMC10084231

[32] Winkelmann J, Gómez Rossi J, Schwendicke F, Dimova A, Atanasova E, Habicht T, et al. Exploring variation of coverage and access to dental care for adults in 11 European countries: a vignette approach. BMC Oral Health. 2022;22:65. 35260137 10.1186/s12903-022-02095-4 PMC8905841

[33] Shora L, Friberg E, Park LT, DeGennaro R, Hinton I, Zarate CA Jr. Assessing geographic disparities in mental health research participation. Contemp Clin Trials. 2023;131:107244. 37257724 10.1016/j.cct.2023.107244 PMC10526678

[34] Mudaranthakam DP, Gajewski B, Krebill H, Coulter J, Springer M, Calhoun E, et al. Barriers to clinical trial participation: comparative study between rural and urban participants. JMIR Cancer. 2022;8:e33240. 35451964 10.2196/33240 PMC9073606

[35] Ministry of internal affairs. and communications: information and communications in Japan, WHITE PAPER 2024, Sect. 11, P149-150. Trends in Digital Usage. https://www.soumu.go.jp/johotsusintokei/whitepaper/eng/WP2024/2024-index.html. Accessed 18 August 2025.

[36] Karniouchina EV, Moore WL, van der Rhee B, Verma R. Issues in the use of ratings-based versus choice-based conjoint analysis in operations management research. Eur J Oper Res. 2009;197:340–8.

[37] Hoffman S, Signorini G, Simons CT. Comparison of ratings based and choice based approaches at integrating sensory attributes with extrinsic product attributes. Food Res Int. 2025;220:117170. 41074379 10.1016/j.foodres.2025.117170

