# Supplementary material for: Preferred conditions for promoting participation in on-site oral health surveys among Japanese adults: insights from a conjoint analysis
Source: BMC Public Health. 2026 Apr 6;26:1583. doi: 10.1186/s12889-026-26736-3 (PMC13192037; doi:10.1186/s12889-026-26736-3)
Supplement: Supplementary file 1 — Supplementary Material 1. [file 12889_2026_26736_MOESM1_ESM.pdf]

Supplementary Materials

■Information for participants (1)

- One of the surveys conducted by the Ministry of Health, Labour and Welfare is the Survey of Dental Disease. The purpose of this survey is to understand the dental and oral health status of people living in Japan and collect basic data for developing dental health policies.
- To conduct the survey, several regions across Japan were selected and residents in those areas were invited to participate. Selected individuals visit venues such as community centres where dentists conduct dental examinations.
- However, in recent years, the number of participants in this survey has declined, which has become a serious concern.

■Information for participants (2)

- The table below shows the specific conditions for each item, such as the location and day of the week on which the Survey of Dental Diseases will be conducted.
- We will now present a total of 16 tables, each combining different sets of conditions.
- For each set of conditions shown, <u>please select the number that best represents whether you would be willing to participate in the survey.</u>

Attributes	Levels
Location	• Public facilities (community centres, health centres, etc.)<br>• Nearby dental clinics<br>• Commercial facilities (supermarkets, shopping malls, etc.)<br>• Home visits by investigators
Day of the week	• Early weekdays (Monday–Wednesday)<br>• Later weekdays (Thursday and Friday)<br>• Saturday<br>• Sunday
Time required for the survey	• As short as possible<br>• Acceptable even if it takes some time
Advance booking of survey time	• Available<br>• Not available
Explanation of oral health status by dentist after survey	• Provided<br>• Not provided
Small gifts (toothbrushes, etc.)	• Provided<br>• Not provided
Orientation session before the survey	• Held<br>• Not held

■Question (1)

- Under the following conditions, would you be willing to participate in the Survey of Dental Diseases?
- Please select the number that best applies to you.

Attributes	Levels
Location	• Public facilities (community centres, health centres, etc.)
Day of the week	• Early weekdays (Monday–Wednesday)
Time required for the survey	• As short as possible
Advance booking of survey time	• Available
Explanation of oral health status by dentist after survey	• Provided
Small gifts (toothbrushes, etc.)	• Provided
Orientation session before the survey	• Held

0	1	2	3	4	5	6	7	8	9	10
□	□	□	□	□	□	□	□	□	□	□

0, Not willing to participate; 5, Neither willing nor unwilling to participate; 10, Willing to participate

Note: The 16 conditions shown in Table 2 were presented to participants in random order.

Additional File 1. Questionnaire provided to the study participants (In the actual study, participants answered this questionnaire in the Japanese language on the web.)

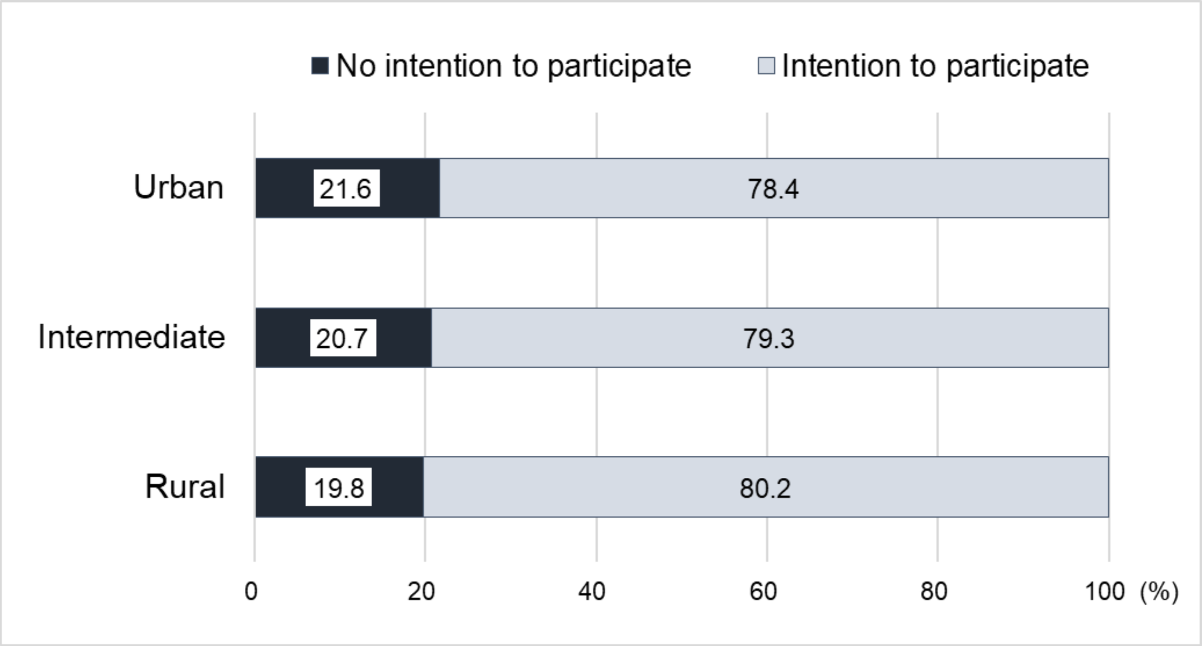

Additional File 2. Proportion of those who did not intend to participate in the oral health survey, classified by municipality type

Additional File 3. Demographic characteristics of the analysis population and the population excluded from the analysis, classified by municipality type

	Urban					Intermediate					Rural				
	analysis population (n=324)		population excluded from the analysis (n=96)		p-Value	analysis population (n=323)		population excluded from the analysis (n=87)		p-Value	analysis population (n=298)		population excluded from the analysis (n=122)		p-Value
	n	(%)	n	(%)		n	(%)	n	(%)		n	(%)	n	(%)	
Sex					0.104					0.399					0.132
Male	155	(47.8)	55	(57.3)		163	(48.9)	47	(54.0)		142	(47.7)	68	(55.7)	
Female	169	(52.2)	41	(42.7)		170	(51.1)	40	(46.0)		156	(52.3)	54	(44.3)	
Age					0.050					0.244					0.261
20–29 years	69	(21.3)	15	(15.6)		69	(20.7)	15	(17.2)		64	(21.5)	20	(16.4)	
30–39 years	70	(21.6)	14	(14.6)		63	(18.9)	21	(24.1)		65	(21.8)	19	(15.6)	
40–49 years	65	(20.1)	19	(19.8)		73	(21.9)	11	(12.6)		55	(18.5)	29	(23.8)	
50–59 years	65	(20.1)	19	(19.8)		65	(19.5)	19	(21.8)		59	(19.8)	25	(20.5)	
60–69 years	55	(17.0)	29	(30.2)		63	(18.9)	21	(24.1)		55	(18.5)	29	(23.8)	
Household income					0.243					0.063					0.304
JPY <2 million	27	(8.3)	9	(9.4)		21	(6.3)	10	(11.5)		27	(9.1)	18	(14.8)	
JPY 2–4 million	49	(15.1)	21	(21.9)		61	(18.3)	11	(12.6)		53	(17.8)	15	(12.3)	
JPY 4–6 million	52	(16.0)	21	(21.9)		63	(18.9)	22	(25.3)		71	(23.8)	23	(18.9)	
JPY 6–8 million	40	(12.3)	8	(8.3)		48	(14.4)	6	(6.9)		31	(10.4)	13	(10.7)	
JPY ≥8 million	60	(18.5)	17	(17.7)		55	(16.5)	10	(11.5)		36	(12.1)	14	(11.5)	
Unknown	96	(29.6)	20	(20.8)		85	(25.5)	28	(32.2)		80	(26.8)	39	(32.0)	
Marital status					0.489					0.296					0.392
Married	175	(54.0)	48	(50.0)		193	(58.0)	45	(51.7)		182	(61.1)	69	(56.6)	
Single	149	(46.0)	48	(50.0)		140	(42.0)	42	(48.3)		116	(38.9)	53	(43.4)	
Working status					0.227					0.244					0.362
Regular worker	178	(54.9)	48	(50.0)		189	(56.8)	39	(44.8)		167	(56.0)	66	(54.1)	
Homemaker	54	(16.7)	13	(13.5)		38	(11.4)	14	(16.1)		41	(13.8)	14	(11.5)	
Part-time worker	54	(16.7)	16	(16.7)		57	(17.1)	19	(21.8)		51	(17.1)	18	(14.8)	
Unemployed/others	38	(11.7)	19	(19.8)		49	(14.7)	15	(17.2)		39	(13.1)	24	(19.7)	
Frequency of brushing teeth					0.124					0.794					0.245
≥Three times daily	81	(25.0)	14	(14.6)		86	(25.8)	23	(26.4)		71	(23.8)	24	(19.7)	
Twice daily	176	(54.3)	57	(59.4)		172	(51.7)	41	(47.1)		159	(53.4)	60	(49.2)	
Once daily	54	(16.7)	18	(18.8)		68	(20.4)	20	(23.0)		55	(18.5)	28	(23.0)	
Sometimes /No brushing	13	(4.0)	7	(7.3)		7	(2.1)	3	(3.4)		13	(4.4)	10	(8.2)	
Regular dental check-up					0.029					0.007					0.002
Yes	166	(51.2)	37	(38.5)		169	(50.8)	30	(34.5)		155	(52.0)	43	(35.2)	
No	158	(48.8)	59	(61.5)		164	(49.2)	57	(65.5)		143	(48.0)	79	(64.8)	

Note: Chi-squared test.

Additional File 4. Values of each attribute and level in the conjoint analysis, classified by municipality type

Attributes	Levels	Urban							Intermediate							Rural						
		coefficient	95% confidence interval		weighted mean	part-worth utility values	part-worth utility range	relative importance	coefficient	95% confidence interval		weighted mean	part-worth utility values	part-worth utility range	relative importance	coefficient	95% confidence interval		weighted mean	part-worth utility values	part-worth utility range	relative importance
Location	Public facilities	0.000			-0.096	0.096	1.135	43.44	0.000			0.034	-0.034	1.177	47.64	0.000			-0.097	0.097	1.084	46.46
	Nearby dental clinics	0.383	0.108	0.657		0.479			0.596	0.396	0.797		0.563			0.435	0.242	0.629		0.532		
	Commercial facilities	-0.016	-0.291	0.258		0.080			0.119	-0.081	0.320		0.086			-0.174	-0.368	0.019		-0.078		
	Home visits by investigator	-0.752	-1.027	-0.478		-0.656			-0.581	-0.782	-0.381		-0.615			-0.648	-0.842	-0.455		-0.552		
Day of the week	Early weekdays	0.000			0.193	-0.193	0.435	16.66	0.000			0.136	-0.136	0.363	14.70	0.000			0.095	-0.095	0.284	12.15
	Later weekdays	-0.014	-0.288	0.260		-0.207			-0.005	-0.206	0.195		-0.141			-0.049	-0.243	0.144		-0.145		
	Saturday	0.421	0.147	0.696		0.228			0.358	0.158	0.559		0.222			0.196	0.003	0.390		0.101		
	Sunday	0.364	0.090	0.639		0.171			0.190	-0.011	0.390		0.054			0.234	0.041	0.428		0.139		
Time required for the survey	As short as possible	0.231	0.037	0.425	0.116	0.116	0.231	8.86	0.212	0.070	0.354	0.106	0.106	0.212	8.58	0.194	0.057	0.331	0.097	0.097	0.194	8.31
	Acceptable even if it takes some time	0.000				-0.116			0.000				-0.106			0.000				-0.097		
Advance booking of survey time	Available	0.076	-0.118	0.270	0.038	0.038	0.076	2.92	0.084	-0.058	0.225	0.042	0.042	0.084	3.39	0.015	-0.122	0.152	0.008	0.008	0.015	0.65
	Not available	0.000				-0.038			0.000				-0.042			0.000				-0.008		
Explanation by dentist	Provided	0.255	0.061	0.449	0.128	0.128	0.255	9.78	0.386	0.245	0.528	0.193	0.193	0.386	15.63	0.360	0.223	0.497	0.180	0.180	0.360	15.43
	Not provided	0.000				-0.128			0.000				-0.193			0.000				-0.180		
Small gifts	Provided	0.353	0.159	0.547	0.177	0.177	0.353	13.53	0.235	0.093	0.376	0.117	0.117	0.235	9.49	0.332	0.195	0.469	0.166	0.166	0.332	14.24
	Not provided	0.000				-0.177			0.000				-0.117			0.000				-0.166		
Orientation session	Held	-0.126	-0.320	0.068	-0.063	-0.063	0.126	4.81	-0.014	-0.156	0.128	-0.007	-0.007	0.014	0.56	-0.065	-0.201	0.072	-0.032	-0.032	0.065	2.77
	Not held	0.000				0.063			0.000				0.007			0.000				0.032		

Additional File 5. Characteristics of those who did not intend to participate in the oral-health survey, classified by municipality type (univariate modified Poisson regression analysis)

	Urban	Intermediate	Rural
	PR (95%CI)	PR (95%CI)	PR (95%CI)
Gender			
Male	1.09 (0.72–1.65)	0.96 (0.63–1.46)	1.39 (0.88–2.21)
Female	reference	reference	reference
Age			
20–29 years	reference	reference	reference
30–39 years	1.23 (0.62–2.44)	0.88 (0.37–2.08)	1.48 (0.72–3.04)
40–49 years	0.80 (0.36–1.77)	1.51 (0.74–3.11)	1.16 (0.52–2.59)
50–59 years	1.15 (0.57–2.34)	1.80 (0.89–3.65)	1.19 (0.55–2.61)
60–69 years	2.20 (1.19–4.06) *	1.97 (0.98–3.95)	1.51 (0.72–3.18)
Household income			
JPY <2 million	reference	reference	reference
JPY 2–4 million	1.65 (0.49–5.61)	0.54 (0.27–1.05)	1.02 (0.47–2.23)
JPY 4–6 million	1.73 (0.52–5.78)	0.41 (0.20–0.85) *	0.65 (0.29–1.48)
JPY 6–8 million	2.70 (0.84–8.69)	0.39 (0.17–0.87) *	0.87 (0.35–2.17)
JPY ≥8 million	2.10 (0.66–6.72)	0.38 (0.18–0.83) *	0.43 (0.14–1.32)
Unknown	2.06 (0.67–6.38)	0.49 (0.26–0.94) *	0.72 (0.33–1.59)
Marital status			
Married	0.95 (0.63–1.45)	0.89 (0.58–1.36)	1.00 (0.62–1.60)
Single	reference	reference	reference
Working status			
Regular worker	reference	reference	reference
Homemaker	0.98 (0.54–1.79)	0.39 (0.13–1.21)	0.84 (0.40–1.76)
Part-time worker	1.51 (0.93–2.47)	1.22 (0.71–2.09)	0.77 (0.38–1.56)
Unemployed/others	0.63 (0.27–1.51)	1.42 (0.84–2.41)	1.26 (0.68–2.33)
Frequency of brushing teeth			
≥Three times daily	0.72 (0.36–1.46)	0.75 (0.42–1.34)	1.00 (0.40–2.51)
Twice daily	1.05 (0.59–1.85)	0.68 (0.41–1.13)	1.83 (0.86–3.87)
Once daily	reference	reference	reference
Sometimes/No brushing	1.38 (0.53–3.61)	1.62 (0.63–4.16)	3.63 (1.46–9.00) **
Regular dental check-up			
Yes	0.80 (0.53–1.22)	0.70 (0.46–1.08)	0.59 (0.37–0.95) *
No	reference	reference	reference

Note: * $p<0.05$, ** $p<0.01$, PR = Prevalence ratio; 95%CI = 95% confidence interval.
